# Dr. Eugene A. Smith Tells of Appendicitis Being Discussed in the European Centers

**Published:** 1906-09

**Authors:** Eugene A. Smith

**Affiliations:** Berne


### CORRESPONDENCE.

### Dr. Eugene A. Smith Tells of Appendicitis Being Discussed in the European Centers.

*Editor Buffalo Medical Journal.*

SIR:—It is interesting to note the culmination of the discussion of appendicitis, which has been warmly under way in European centers of medical teaching for some months. It reminds an American surgeon of the public and scientific discussion of this disease during the past ten years at home, culminating two or three years ago in general aquiscence in its surgical treatment.

In Great Britain and on the continent treatment of appendicitis is now changing from the ultra conservative or non-operative to the radical, or to use a better phrase, the surgical treatment. Moynihan in England, Hartman in Paris, and Kocher in Berne, all of whom I met, are the advocates of early diagnosis and early operation. A German army surgeon, an aberstabs arzt, told me that cases of appendicitis are now frequently brought to early operation in the German army, which is doing very well for Germans who formerly called appendicitis the American disease.

That the laity have also reached a certain phase of education in the pathology of the disease is shown by incidents coming under my observation. On the train lately an English lady of education cautioned her ten-year-old son, to whom I gave an orange, not to eat the seeds for fear they might cause appendicitis. The innkeeper at Berne, not knowing my profession, in talking of hygiene in general prompted by my inquiry as to the Berne drinking water, gravely said, "It is a pity one cannot now eat cherries whole as we did formerly, because of fear of death from appendicitis."

The pathology of appendicitis is discussed here as we discuss it,—namely, as a primary bacterial disease with various predisposing causes. But the need of a close study of its symptomatology with a view to early differential diagnosis from other abdominal diseases is not generally well recognised. It follows that the majority of cases of appendicitis coming for operation are late cases, with perforation, gangrene, general peritonitis or localised abscess. Several operators told me this is the fact.

I saw five cases of appendicitis in Kocher's wards while at Berne. Two were operated upon early without drainage. Two were drained as pus cases. One came to post mortem because of general peritonitis due to perforated appendix, abdominal incision with multiple drains having been vainly tried immediately on reception of the patient.

In a well known English clinic I saw a case of appendicitis treated by incision and drainage of an abscess in the iliac fossa. The patient had entered the hospital two weeks before in the first twenty-four hours of acute abdominal illness, it being the second attack of the same kind. Typhilitis was diagnosed on his entry. Pulse, temperature, respiration, blood count and clinical history plainly indicated acute appendicitis. Operation finally became imperative after two weeks of waiting for an interval operation, and of course was far more hazardous than early operation would have been. In another clinic, under the observation of a famous surgeon, a boy of fifteen entered the out-patient department with a history of eighteen hours of acute abdominal pain, rapid pulse, and high temperature, and with a history of a similar attack eight months previously. A diagnosis of appendicitis without perforation or pus formation was made and he was sent to the ward to await developments, preparation for operation being ordered if the symptoms became more urgent in the next few hours. Marked improvement soon developed and on the third day he was discharged as cured without operation.

At a crowded meeting of the Berlin Medical Society last week appendicitis was discussed by many of the leading German surgeons including Israel, Krause, Landau, and Olshausen. All were agreed that a certain percentage of cases of appendicitis pass through one or more attacks without the need of an operation, and if relapse does not occur within two years of a first attack, the likelihood of a second seizure is small. But this consolation, if it is one for nonoperative treatment, is disturbed by the fact that 50 per cent. of patients having an attack have a second one within a year. To sum up a long discussion, it was agreed that a prognosis cannot be given as to a favorable or unfavorable termination of an attack in a given case and, therefore, the early operative treatment is the safest.

On several occasions quite flattering commendations of the advances made by American medicine and American surgery were made to me by men whose opinions we respect. But Kocher gave us the most sincere praise in a lecture to the surgeons of the Swiss army corps in their annual course, which I had the pleasure of attending during the last ten days. Lecturing on appendicitis, he dwelt on American work and illustrated his lecture by exhibiting the plates from Kelly's book on this disease.

While it seems to me that American surgical thought and method are distinctly in advance of the European in the discussion and treatment of appendicitis, I do not wish to be considered as saying we have reached the end of our endeavors. We still have to convince many medical men by repeated proof that early operation is the treatment for this disease and we still have some who are dilatory, if not negligent, in the early differential diagnosis of appendicitis. The majority of American medical men, however, are agreed on this subject, but in Europe only the leaders seem agreed, while the rank and file are apparently still to be convinced and educated.

EUGENE A. SMITH.

BERNE, AUGUST 1, 1906.

